# Liver Cyst Infection Outcomes in Patients With ADPKD

**DOI:** 10.1016/j.ekir.2025.10.027

**Published:** 2025-11-04

**Authors:** Charles Ronsin, François Jouret, Simon Ville, Jihad Abdelmalki, Grégoire Couvrat-Desvergnes, Léo Drapeau, Raphael Gaisne, Benjamin Gaborit, Caroline Charlier, Mohamad Zaidan, Renaud Snanoudj, Magali Giral, Jacques Dantal, Bertrand Knebelmann, Julien Dang

**Affiliations:** 1 Department of Nephrology and Immunology, Nantes University Hospital, Nantes, France; 2 Division of Nephrology, Department of Internal Medicine, University of Liège Hospital, Liège, Belgium; 3 Laboratory of Translational Research in Nephrology, GIGA Institute (Metabolism and Cardiovascular Biology), University of Liège, Liège Belgium; 4 Transplantation and Immunology Research Center; UMR 1064, INSERM, Nantes University, Nantes, France; 5 Department of Nephrology, Dialysis, and Transplantation, Departmental Hospital of Vendée, La Roche-sur-Yon, France; 6 Department of Nephrology, Saint Nazaire Hospital, Saint Nazaire, France; 7 Department of Infectious Diseases, Nantes University Hospital, Nantes, France; 8 Department of Infectious Diseases, Cochin University Hospital, Paris, France; 9 Department of Nephrology and Transplantation, Bicêtre Hospital, Le Kremlin-Bicêtre, France; 10 Department of Nephrology and Transplantation, Paris Cité University, Necker-Enfants Malades Hospital, Paris, France; 11 Department of Nephrology and Dialysis, Ambroise Paré Hospital, Boulogne-Billancourt, France

**Keywords:** autosomal dominant polycystic kidney disease, cyst infection, liver cyst

## Abstract

**Introduction:**

Liver cyst infection is a rare and severe complication of the liver cysts associated with autosomal dominant polycystic kidney disease (ADPKD), and evidence-based data for optimal management is lacking. We conducted a multicentric retrospective study to investigate the treatment and outcomes of liver cyst infection.

**Methods:**

Liver cyst infection was either defined by (i) C-reactive protein levels ≥ 50 mg/l and suspicion at computed tomography (CT) scan, ^18^Fluorodeoxyglucose (^18^FDG) positron-emission tomography (PET) CT, magnetic resonance imaging (MRI); or (ii) proven by cyst puncture. We studied the determinants of treatment failure (persistent infection with requirement for antibiotic therapy change, cyst drainage, and hepatectomy), relapse (< 2 months) and recurrence (> 2 months) of liver cyst infection after antibiotics discontinuation.

**Results:**

Sixty-two patients and 112 episodes were included. At least 1 microorganism was identified in 70 of 112 episodes (63%), mainly *Escherichia coli* in 36 of 70of cases (51%). *E coli* was resistant to third generation cephalosporin, fluoroquinolone, or cotrimoxazole in 13%, 16%, and 34%, respectively. Treatment failure and relapse occurred in 30 of 112 episodes (27%). Antibiotic therapy duration ≥ 14 days was a protective factor for treatment failure or relapses (odds ratio [OR] = 0.03, 95% confidence interval [CI]: 0–0.23], *P* = 0.006). Recurrence occurred in 24 of 62 patients (38%), within 1 year for 15 patients (24%) after the first episode. An antibiotic therapy duration ≥ 28 days was identified as a protective factor (OR = 0.12, 95% CI: 0.02–0.65], *P* = 0.021). Conversely, a history of renal cyst infection significantly increased the risk of recurrence within 1 year (OR = 9.22 95% CI: 1.28–99.55], *P* = 0.04).

**Conclusion:**

Treatment failure or relapse or recurrence of liver cyst infection both occurred in one-third of cases, and are associated with a shorter antibiotic therapy duration < 28 days.

## Graphical abstract

**Figure:**
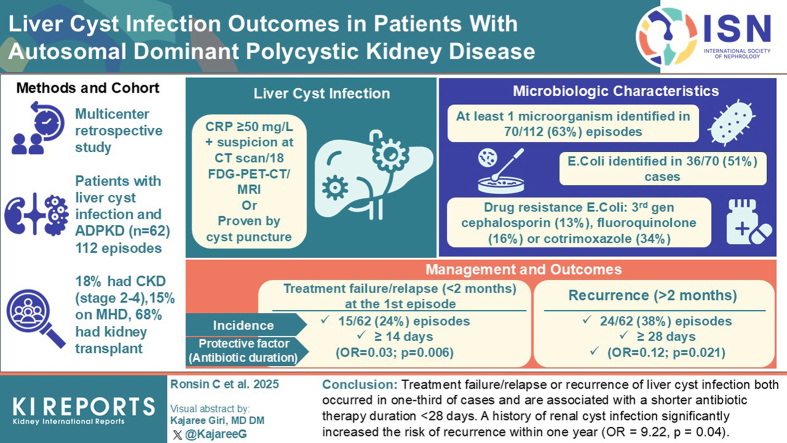

ADPKD is the most common inherited kidney disease, characterized by the occurrence of numerous renal cysts and kidney enlargement, with progressive loss of renal function and eventual end-stage renal disease.^1^ Apart from the kidney, ADPKD can affect multiple organs, with polycystic liver disease being the most common extrarenal complication.^2^ Liver cysts are lined by epithelial cells with phenotypic and functional characteristics of biliary epithelium with enhanced secretory and proliferative activities.^3^ These liver cysts are present in > 90% of patients aged > 35 years with ADPKD^4^ and polycystic liver disease tends to be more severe in females with exogenous estrogen exposure or multiple pregnancy.^1^ Although most patients with liver cysts remain asymptomatic, a minority will develop complications such as extrinsic obstruction of the adjacent structure, cyst hemorrhage, or cyst infection. Liver cyst infection is one of the most challenging complications of polycystic liver disease, occurring in ≤ 3% of patients with ADPKD.^5^ Clinical symptoms of liver cyst infection can range from an isolated fever and abdominal pain to more serious complications such as treatment failure, recurrent or chronic infection, and death.^6^^,^^7^

Despite differences in pathophysiology and causative microorganisms,^8^ the management of liver cyst infection is often modeled on renal cyst infection, which has been extensively studied.^6^^,^^9^, ^10^, ^11^, ^12^ Although formal evidence on the type and the adequate duration of antibiotic therapy is lacking, clinical guidelines suggest ≥ 4 weeks of antibiotic therapy with longer treatment period on a case-by-case basis (European Association for the Study of the Liver^13^ and Kidney Disease: Improving Global Outcomes 2025^14^).^12^ However, treatment failure occurs in ≤50%,^15^ and approximately 10% of patients will die from uncontrolled infections in a Japanese monocentric study, consistent with a case-based review reported by Lantinga (9% of 54 patients).^7^^,^^15^^,^^16^ In addition, liver cyst infection recurrences are common (in 14%–50% of cases)^7^^,^^17^ and associated with significant morbidity such as multiple hospitalizations, malnutrition, and invasive procedures such as partial hepatectomy. Therefore, evidence-based data on the optimal treatment modalities of liver cyst infection are needed. For this purpose, we conducted a retrospective multicenter study to assess the factors associated with treatment failure, relapse, and recurrence of liver cyst infection in patients with ADPKD.

## Methods

### Study Population

Patients with liver cyst infection and ADPKD were identified retrospectively through computerized medical records from January 2012 to May 2024 in 4 referral centers (3 in France and 1 in Belgium). Data were collected retrospectively using an anonymized standardized case report form. This study was conducted in accordance with the Declaration of Helsinki and was approved by the institutional review boards at each participating site. Given the retrospective nature of the study, written informed consent was not required in France or Belgium. Information letters about this study were provided to all patients included in the series as requested in France.

#### Definitions

Liver cyst infection was defined as follows: (i) a C-reactive protein ≥ 50 mg/l^6^ and suggestive signs at on CT scan, MRI, or ^18^FDG PET/CT and/or (ii) the presence of microorganism and/or neutrophil debris proven by cyst puncture.^6^^,^^18^ Positive CT scan or MRI for liver cyst infection was defined by cyst wall thickening, with contrast enhancement in case of injection, and/or peri-cystic fat infiltration. Positive ^18^FDG-PET/CT for liver cyst infection was defined by a focally increased ^18^FDG uptake around or inside ≥1 liver cysts.^19^ Treatment failure was defined as the persistence of clinical or biochemical signs of infection (fever, sepsis, no decrease of C-reactive protein level and/or positive microbial cultures) beyond 72 hours of microbiologically adequate antibiotic therapy that would require increasing doses of antibiotic, antibiotic change, cyst drainage, or partial hepatectomy. Relapse was defined as the reappearance of clinical symptoms of liver cyst infection <2 months after antibiotic discontinuation (same microorganism as in the index episode if documented).^20^ Recurrence was defined as a new episode of liver cyst infection >2 months after antibiotic discontinuation.^20^ Severe sepsis was defined by sepsis and systolic blood pressure < 90 mm Hg, and septic shock was defined as sepsis and the need for catecholamines administration.

### Statistical Analysis

Continuous variables were described using mean values with SD or median values with interquartile range as appropriate, whereas categorical variables were given as counts and percentages. We first conducted univariable analyses using Fisher exact test for binary variables and Wilcoxon’s rank-sum test for continuous variables to identify factors associated with treatment failure or relapse at the first episode of liver cyst infection. Variables with a *P*-value < 0.20 in univariable analysis were then entered into a multivariable logistic regression model. A stepwise backward and forward selection procedure based on Akaike’s Information Criterion was used to identify the final model. A *P*-value < 0.05 was considered statistically significant. Multivariable analysis was performed using generalized linear models with a logit link. Treatment failure and relapse were analyzed in conjunction because they are both early outcomes of the initial management. Only the first episode of each patient was considered for analyses to avoid index event bias.^21^ Statistical analyses and the cumulative incidence curve of recurrent liver cyst infection were performed or obtained using R software version 4.4.0, dplyr, epitools, survival and ggplot2 packages.

## Results

### Clinical, Biological, and Imaging Features

Sixty-two patients had 112 episodes of liver cyst infection. Eighteen episodes (16%) were proven by cyst puncture (at diagnosis or after a cyst drainage for treatment failure). At the time of the first liver cyst infection episode, the mean age was 64 ± 9.6 years, 39 (63%) were female, 11 (18%) had CKD stage 2 to 4, 9 (15%) were on maintenance dialysis ,42 patients (68%) had a kidney transplant, and none were on somatostatin analogue (Table 1).

**Table 1:** Characteristics of the 62 patients with ADPKD with 112 episodes of liver cyst infection

Features of liver cyst infection at diagnosis	Liver cyst infection Patients, *n* = 62 Episodes, *n* = 112
Characteristics of patients at their first episode of liver cyst infection	*n* = 62
Demographic characteristics	
Age (yr)	64 ± 9.6
Female	39 (63%)
Past medical history	
Diabetes	9 (15%)
Cholecystectomy	9 (15%)
Diverticulitis	12 (19%)
Malignancy (excluding non-melanoma skin cancer)	7 (11%)
Kidney transplant	42 (68%)
Maintenance dialysis	9 (15%)
Chronic kidney disease, stages 2-4	11 (18%)
Use of antibiotic therapy within the previous year^a^	28 (45%)
Characteristics of the episodes of liver cyst infection	*n* = 112
Diagnosis	
Cyst puncture	18 (16%)
Imaging	
Positive rate of CT scan	23/76 (30%)
Positive rate of ^18^FDG-PET/CT	94/98 (96%)
Positive rate of MRI	2/2 (100%)
Clinical features	
Fever	92 (82%)
Abdominal pain	63 (56%)
Isolated fever	34 (30%)
Sepsis severe/septic shock	9/70 (13%)
Biological features	
C-reactive protein (mg/l)	197 ± 96
Leukocyte count (cells/mm^3^)	9293 ± 4798^b^
*De novo* serum aminotransferases increase	15/107 (14%)^b^
*De novo* alkaline phosphatase +gamma glutamyl transferase increase	36/107 (34%)^b^
Acute kidney injury	42/103 (41%)
Imaging features on positive ^18^FDG-PET/CT	
Multiple infected cysts	61/94 (65%)
Concomitant renal cyst infection	3/94 (3%)
Microbiological features	
Positive rate	70/112 (63%)
Blood culture	56/70 (80%)
Cyst fluid culture	5/70 (7%)
Blood and cyst fluid culture	9/70 (13%)
Microorganism(s)	
Polymicrobial	5/70 (7%)^c^
Gram negative	62/70 (89%)
*E. coli*	36/70 (51%)
*Pseudomonas* species	7/70 (10%)
*Acinetobacter* species	2/70 (3%)
Gram positive^d^	8/70 (11%)
Fungi^e^	1/70 (1%)
Antibiotic resistance of *E. coli*	
Missing data	4/36 (11%)
Fluoroquinolone	5/32 (16%)
3rd generation cephalosporin	4/32 (13%)
Trimethoprim-sulfamethoxazole	11/32 (34%)
Amoxicillin/clavulanic acid	6/32 (19%)
Piperacillin/tazobactam	3/32 (9%)

^18^FDG, ^18^Fluorodeoxyglucose; ADPKD, autosomal dominant polycystic kidney disease; CT, computed tomography; MRI, magnetic resonance imaging; PET, positron emission tomography.

Continuous variables were described as mean and SD, and categorical variables were described as counts and percentages.

a Including sulfamethoxazole-trimethoprim prophylaxis in kidney transplant patients.

b Missing data for white blood cell count, *n* = 3; for liver test, *n* = 5.

c *Pseudomonas aeruginosa* + *Enterobacter cloacae*, *Escherichia coli* + *Pseudomonas aeruginosa* + *Pseudomonas fluorescens*, *E coli* + *Enterococcus faecium*, *E coli* + *Citrobacter freundii* + *Klebsiella pneumoniae*, and *E coli* + *Acinetobacter* species.

d *Staphylococcus epidermidis* (*n* = 2), *Staphylococcus hominis* (*n* = 1), *Enterococcus faecium* (*n* = 2), *Enterococcus gallinarum* (*n* = 1), *Lactobacillus* spp. (*n* = 1), *Streptococcus pneumoniae* (*n* = 1).

e *Candida glabrata*.

The main symptoms and laboratory data are shown in Table 1. Abdominal pain and fever were absent in 49 (44%) and 20 (18%) episodes, respectively. Severe sepsis or septic shock occurred in 15 episodes (13%). Laboratory data showed leukocytosis in 48 episodes (43%) with a mean leukocyte count at 9293 ± 4798/mm^3^, mean C-reactive protein at 197 ± 96 mg/l. *De novo* alkaline phosphatase + gamma glutamyl transferase increase or serum aminotransferases increase occurred in 36 of 107 (34%) and 15 of 107 (14%), respectively; and acute kidney injury occurred in 42 of 103 episodes (41%) (35 AKIN 1, 3 AKIN 2, and 4 AKIN 3). ^18^FDG-PET/CT was performed in 98 episodes (suggestive of liver cyst infection in 94 (96%)), CT scan was performed in 76 episodes, including 54 with contrast enhancing media, and suggestive of liver cyst infection in 23 episodes (30%). MRI was performed in 2 episodes (suggestive of liver cyst infection in both episodes). Among definite episodes of liver cyst infection, ^18^FDG-PET/CT was performed in 14 episodes (suggestive of liver cyst infection in 13 [93%]) and CT scan was performed in 13 episodes (suggestive of liver cyst infection in 5 [39%]) (Supplementary Table S1).

### Microbiological Characteristics

Microbiological data are summarized in Table 1, and more detailed in Supplementary Tables S2 and S3. At least 1 microorganism was identified in 70 of 112 (63%) episodes (56 in blood culture only, 5 in cyst fluid culture only, and 9 in both blood and cyst fluid cultures). Polymicrobial liver cyst infection was documented in 5 of 70 episodes (7%). The 2 most common bacteria were *E coli* (36/70, 56%) and *Klebsiella pneumoniae* (10/70, 14%). Antibiotic susceptibility testing of *E coli* was available in 32 episodes and showed resistance to cotrimoxazole, amoxicillin-clavulanic acid, fluoroquinolone, third-generation cephalosporin and piperacillin/tazobactam in 34%, 19%, 16%, 13%, and 9%, respectively.

A microbial portal of entry was sought in 25 patients (40%). A colonoscopy was performed after a liver cyst infection in 17 episodes and showed polyps in 3 patients. Liver and biliary MRI was performed in 10 patients and showed a suspected malignancy in the hepatic dome in 1 patient (disproved by exploratory laparoscopy), a fistula between hepatic duct and an infected liver cyst in 1 patient, and a mild dilatation of the common bile duct in 2 patients. Diverticulosis was found on CT scan or colonoscopy in 20 of the 55 patients (36%) who underwent a CT scan or a colonoscopy during their follow-up.

### Management and Outcomes

Probabilistic initial therapy (108/112, 96%) consisted of a single antibiotic in 70 of 108 (65%), a dual antibiotic in 34 of 108 (32%), and a triple antibiotic in 4 of 108 (4%) episodes. In 4 episodes, antibiotic therapy was directly adapted to antibiotic susceptibility testing and no probabilistic antibiotic therapy was initiated. Beta-lactam was the most used in monotherapy in 67 of 108 of cases (62%), including third generation cephalosporin in 31 of 108 (29%), fourth generation cephalosporin in 2 of 108 (2%), piperacillin/tazobactam in 17 of 108 (16%), carbapenem in 8 of 108 (7%), oxacillin in 1 of 108 (1%), and amoxicillin/clavulanic-acid in 8 of 108 (8%). Dual antibiotic regimen included beta-lactam + aminoglycosides in 13 of 108 (12%), beta-lactam + metronidazole in 13 of 108 (12%), and beta-lactam + fluoroquinolone in 3 of 108 (3%), and other dual antibiotic in 5 of 108 (5%) (Table 2). Final antibiotic therapy (after microbiological culture result and antibiotic susceptibility testing) mainly consisted of a single antibiotic in 94 of 112 (84%), with the use of beta-lactam in 54 of 112 (48%), fluoroquinolone in 34 of 112 (30%) or other antibiotic in 6 of 112 (5%) episodes. Final antibiotic therapy was administered via oral route, i.v. route, or as a combination of both (dual therapy: 1 oral and 1 i.v. agent) in 64, 43, and 5 episodes, respectively. Treatment failure or relapse rate were comparable between episodes treated orally (16/64, 25%) and those treated i.v. (12/43, 28%; *P* = 0.83). The median duration of antibiotic therapy was 40 (28–42) days and 25 episodes (22%) were treated with < 28 days of antibiotic therapy.

**Table 2:** Treatment and outcomes of 112 episodes of liver cyst infections occurring in 62 patients with ADPKD

Treatment and outcomes of liver cyst infection	Liver cyst infection Patients, *n* = 62; Episodes, *n* = 112
Treatment	
Initial probabilistic antibiotic therapy	108/112 (96%)
Single molecule	70/108 (65%)
Beta-lactam	67/108 (62%)
Third generation cephalosporin	31/108 (29%)
Fourth generation cephalosporin	2/108 (2%)
Amoxicillin/clavulanic acid	8/108 (7%)
Oxacillin	1/108 (1%)
Piperacillin/tazobactam	17/108 (16%)
Carbapenem	8/108 (7%)
Fluoroquinolone	2/108 (2%)
Daptomycin	1/108 (1%)
Dual antibiotic therapy	34/108 (31%)
Beta-lactam + aminoglycoside	13/108 (12%)
Beta-lactam + fluoroquinolone	3/108 (3%)
Beta-lactam + metronidazole	13/108 (12%)
Others	5/108 (5%)^a^
Triple antibiotic therapy	4/108 (4%)^b^
Final follow-up antibiotic therapy	112/112 (100%)
Single molecule	94/112 (84%)
Beta-lactam	54/112 (48%)
Fluoroquinolone	34/112 (30%)
Others	6/112 (5%)^c^
Dual antibiotic therapy	17/112 (15%)
Beta-lactam + fluoroquinolone	10/112 (9%)
Beta-lactam + other	3/112 (3%)^d^
Fluoroquinolone + other	4/112 (4%)^e^
Median duration of antibiotic therapy (d)	40 [28-42]
Outcomes	
Per episode	*n* = 112
Treatment failure	16/112 (14%)
Antibiotic therapy escalation	14/112 (13%)
Cyst drainage	5/112 (5%)
Partial hepatectomy	1/112 (1%)
Relapses	18/112 (16%)
Recurrences	50/112 (45%)
Recurrence within 1 yr	29/112 (26%)
Per patient	*n* = 62
≥ 1 recurrence, *n* (%)	24/62 (39%)
≥ 3 recurrences, *n* (%)	7/62 (11%)
≥ 5 recurrences, *n* (%)	2/62 (3%)
Follow-up (yrs)	3 [1.1;5.8]
Death during the follow-up	12/62 (19%)
Death attributed to uncontrolled liver cyst infection	0/62 (0%)

ADPKD, autosomal dominant polycystic kidney disease.

Continuous variables were described as median and interquartile ranges, and categorical variables were described as counts and percentages.

a Beta lactam + linezolid (*n* =1), vancomycin (*n*=2), doxycycline (*n* = 1), and daptomycin + clindamycin (*n* = 1).

b Beta-lactam + Metronidazole + aminoglycoside (*n* = 3) and Beta-lactam + quinolone + aminoglycoside (*n* = 1)

c Sulfamethoxazole + trimethoprime (*n* = 3), linezolid (*n* = 2), and antifungal (*n* = 1).

d Metronidazole (*n* = 1), aminoglycoside (*n* = 1) and tigecyclin (*n* = 1).

e Metronidazole (*n* = 2), sulfamethoxazole + trimethoprime (*n* = 1), and clindamycin (*n* = 1).

**Figure 1:**
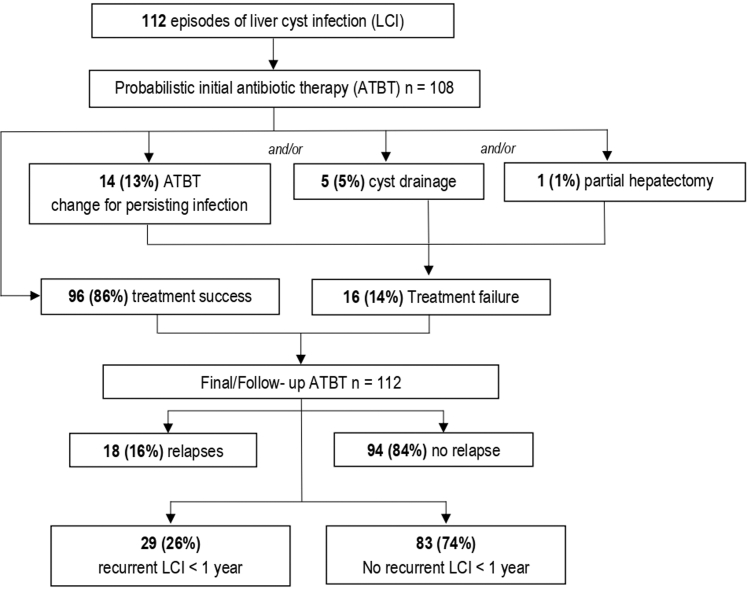
Outcomes of the 112 episodes of liver cyst infection.

**Figure 2:**
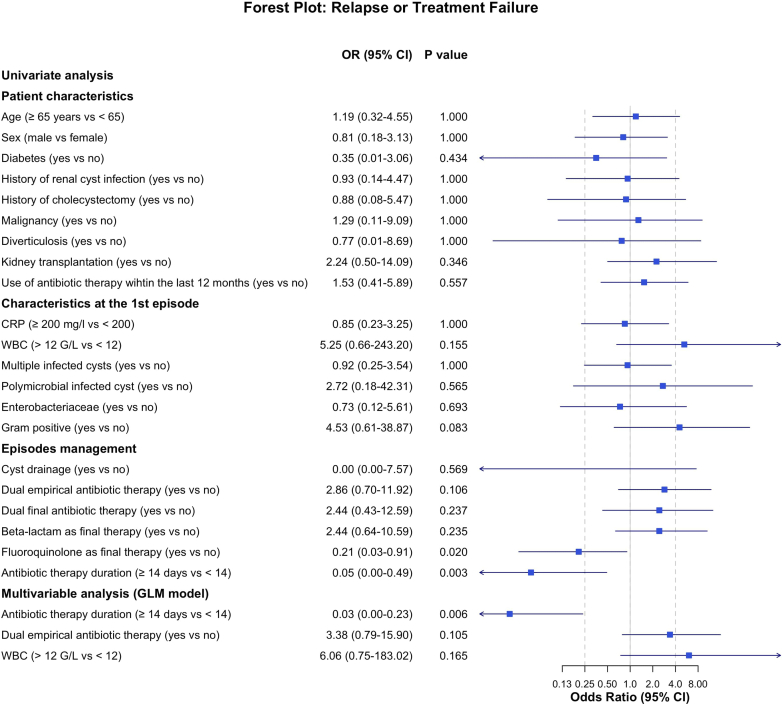
Factors associated with treatment failure or relapse at the first episode of liver cyst infection. Odds ratios and 95% CIs are shown. Variables with *P* < 0.20 in univariable analysis were included in a multivariable logistic regression using stepwise Akaike’s Information Criterion selection. Fluoroquinolone as final therapy was significantly associated with a lower risk of treatment failure or relapse in univariable analysis but was not retained in the final multivariable model. CI, confidence interval; CRP, C-reactive protein; GLM, generalized linear model; WBC, white blood cell.

Treatment failure occurred in 16 of 112 episodes (14%) with antibiotic therapy escalation in 14 of 112 episodes (13%), cyst drainage in 5 of 112 episodes (5%) and partial hepatectomy in 1 of 112 episodes (0.8%). Relapses (within 2 months after antibiotic discontinuation) occurred in 18 of 112 episodes (16%) (Figure 1). Taken together, treatment failure or relapses occurred in 30 of 112 episodes (27%) (4 episodes had both treatment failure and relapses) (Table 2). To identify risk factors for treatment failure and relapses of liver cyst infection, only the first episode was included in the analysis (15/62, 24%). Multivariable analysis identified antibiotic therapy ≥ 14 days (OR = 0.03, 95% CI: 0–0.23, *P* = 0.006) as protective factors for treatment failure or relapse. The use of fluoroquinolone as final antibiotic therapy was statistically protective in univariate analysis (OR = 0.21, 95% CI: 0.03–0.91, *P* = 0.02); however, the association did not persist in multivariable analysis (Figure 2).

Twenty-three patients had a culture-negative liver cyst infection during their first episode. Seven patients received fluoroquinolones (median duration: 28 [25–40] d), 13 received beta-lactams (median duration: 31 [17–37] d), and 3 received other antibiotics as a single molecule regimen. Among patients receiving fluoroquinolones as antibiotic therapy, 0 had treatment failure or relapse, and 1 of 4 patients (25%) with at least 12 months of follow-up had recurrence within a year. Among patients who received beta-lactam as antibiotic therapy, most were kidney transplant recipients (85%), 3 (23%) had treatment failure or relapse, and 5 of 11 patients (46%) with ≥ 1 year of follow-up had recurrence within 12 months (*P* = 0.60).

### Recurrences

**Figure 3:**
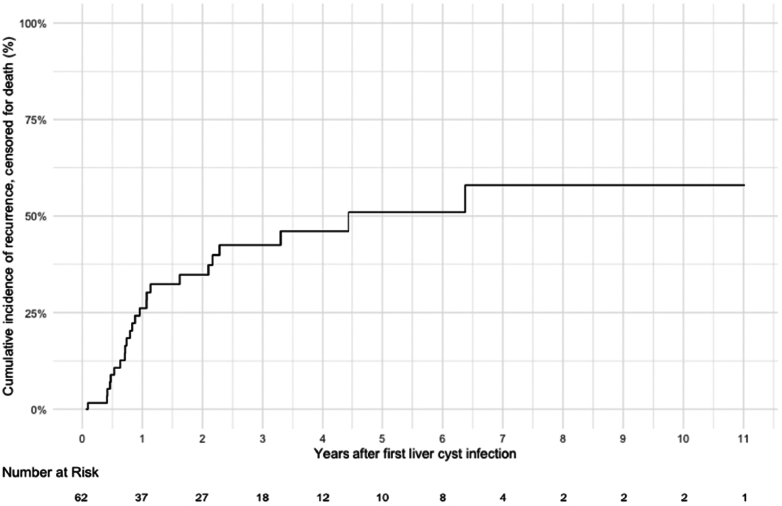
Cumulative incidence, censored at the time of death, of recurrence after a first episode of liver cyst infection.

Overall, 24 of 62 patients (39%) experienced recurrence (after 2 months after antibiotic discontinuation) following their first liver cyst infection after a median follow-up of 3 (1.1–5.8) years. The median delay between the first episode and the first recurrence was 0.8 (0.5–1.7) years. Among all patients, 7 of 62 (11%) had ≥3 episodes and 2 (3%) had ≥5 episodes of recurrence. The cumulative incidence of recurrence increased to 24% and 55% at 1 and 5 years, respectively (Figure 3) and the incidence rate was 10.8 per 100 person-years.

**Figure 4:**
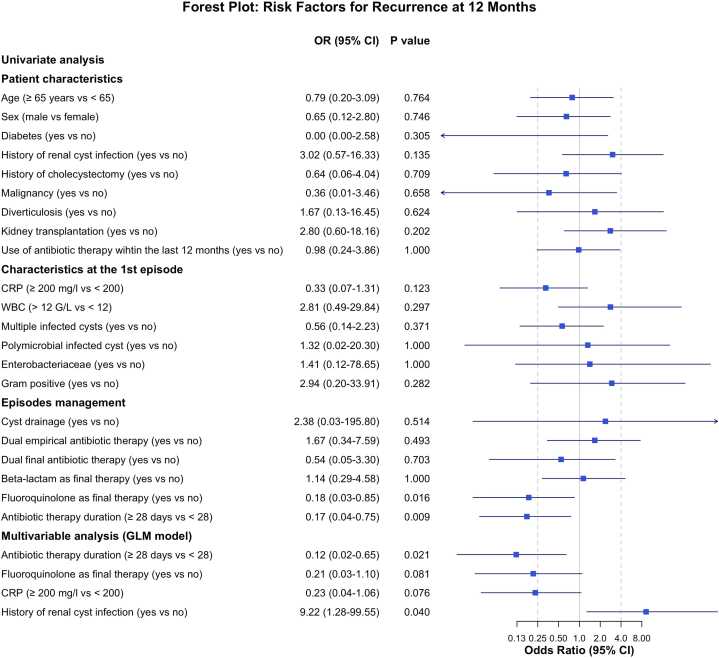
Factors associated with recurrence within 12 months following the first episode of liver cyst infection in 51 patients. Odds ratios and 95% confidence intervals are shown. Variables with p < 0.20 in univariable analysis were included in a multivariable logistic regression using stepwise Akaike’s Information Criterion selection. CI, confidence interval; CRP, C-reactive protein; GLM, generalized linear model; WBC, white blood cell.

To identify risk factors for recurrence within 12 months after a liver cyst infection episode, only the first episode was included in the analysis, and patients with <12 months of follow-up (*n* = 11) were excluded. Multivariable analysis identified an antibiotic therapy duration ≥ 28 days (OR = 0.12, 95% CI: 0.02–0.65, *P* = 0.021) as protective and history of renal cyst infection (OR = 9.22, 95% CI: 1.28–99.55, *P* = 0.004) as risk factors associated with recurrent liver cyst infection (Figure 4).

Follow-up ^18^FDG-PET/CT was performed in 19 episodes (15 patients, Table 3). Fourteen ^18^FDG-PET/CT were performed after discontinuation of antibiotic therapy (median of 22 [7–53] days) and 5 were performed during antibiotic therapy (median of 42 [27–58] days after the start of antibiotic). Follow-up ^18^FDG-PET/CT led to a change in management in 3 patients. One patient with no fever and a mild increase of C-reactive protein (7 mg/l) under ceftolozane/tazobactam for *Pseudomonas aeruginosa*–related liver cyst infection underwent follow-up ^18^FDG-PET/CT 5 days before the originally planned antibiotic therapy discontinuation. The imaging revealed partial diminution of the previous cyst hypermetabolism, and the hypermetabolism persistence of another hypermetabolic cyst. Cyst drainage of the persistent hypermetabolic cyst was performed and showed purulent cyst fluid positive for *P aeruginosa* leading to an additional 6 weeks of antibiotic therapy (Supplementary Figure S1). One patient had antibiotic therapy resumed following persistent cyst hypermetabolism on ^18^FDG-PET/CT, and another patient received 14 additional days of antibiotic therapy. Among the 8 episodes of liver cyst infection with persistent ^18^FDG uptake on liver cyst at follow-up imaging, 2 (25%) had a liver cyst infection relapse (within 2 months after antibiotic discontinuation) after the follow-up ^18^FDG-PET/CT and 4 of 6 (67%) had liver cyst infection recurrence within 12 months. Among them, the median time between follow-up imaging and recurrence was 4 (3–5) months. Of the 11 episodes with no ^18^FDG uptake on liver cyst at follow-up imaging, none had relapse and 3 of 9 (33%) of patients (followed ≥ 1 year after follow-up ^18^FDG-PET/CT) had liver cyst infection recurrence within 12 months. This risk of liver cyst infection recurrence within 12 months among patients with persistent ^18^FDG uptake at follow-up imaging was not statistically significant (*P* = 0.31). When relapses and recurrences (within 12 months) are combined, 6 of 8 (75%) with persistent ^18^FDG cyst uptake versus 3 of 9 (33%) of patients without ^18^FDG cyst uptake developed relapse or recurrence within 12 months after the follow-up imaging (*P* = 0.15) (Table 3).

**Table 3:** Characteristics and outcomes of the 19 episodes of liver cyst infection with baseline and follow-up ^18^FDG-PET/CT

Variables	Complete disappearance of liver cyst uptake *n* = 11	Persistence of liver cyst uptake *n* = 8	*P*-value
Characteristics and outcomes at the index episode			
Age (yrs)	67 ± 11	62 ± 15	0.47
White blood count (cell/mm^3^)	7166 ± 3260	11288 ± 7437	0.18
C-reactive protein (mg/l)	186 ± 60	208 ± 16	0.63
Antibiotic therapy duration (d)	50 ± 19	40 ± 17	0.30
Fluoroquinolone use as final antibiotic therapy	4 (36%)	2/8 (25%)	1
Beta-lactam use as final antibiotic therapy	6 (55%)	6/8 (75%)	0.63
Timing of imaging			
Before antibiotic discontinuation	2 (18%)	3/8 (38%)	0.60
After antibiotic discontinuation	9 (82%)	5/8 (62%)	0.60
Median time between antibiotic therapy start and follow-up imaging (d)	60 [48;79]	74 [45;107]	0.47
At the time of follow-up imaging			
Fever	0	3/8 (38%)	0.06
C-reactive protein (mg/l)	20 ± 21	30 ± 30	0.64
Result of follow-up imaging			
Complete disappearance	11 (100%)	0	
Persistence of ^18^FDG uptake	0	0	
Partial decrease of ^18^FDG uptake	0	4/8 (50%)	
Disappearance of ^18^FDG uptake on initial infected cyst but appearance on another cyst	0	4/8 (50%)	
Outcomes			
Relapses	0/11 (0%)	2/8 (25%)	0.16
Recurrence within 12 mo following imaging^a^	3/9 (33%)	4/6 (67%)	0.31
Relapses or recurrence within 12 mo^b^	3/9 (33%)	6/8 (75%)	0.15
Median time between follow-up imaging and recurrence (mo)	11 [7–20]	6 [4–16]	0.99

^18^FDG, ^18^Fluorodeoxyglucose; ADPKD, autosomal dominant polycystic kidney disease; CT, computed tomography; PET, positron emission tomography.

Continuous variables were described as mean and SD or median and interquartile range, and categorical variables were described as counts and percentages.

a Patients with < 12 months of follow-up after the follow-up ^18^FDG PET/CT and those with relapse prior to recurrence were excluded from the analysis.

b Patients with < 12 months of follow-up after the follow-up ^18^FDG PET/CT were excluded from the analysis.

A prophylactic measure to prevent recurrence of liver cyst infection was performed in 14 patients. Eight patients received long-term antibiotic prophylaxis or cycling antibiotics (Supplementary Table S4). Of these, 2 had recurrences of liver cyst infection (2 and 4 months after the start of antibiotic prophylaxis/cycling). The 6 other patients had a median follow-up of 7 (4.9–11) months (mean: 17.5 ± 27) after starting the prophylaxis. One patient received i.v. Ig but developed a liver cyst infection 11 months later. Six patients underwent invasive procedures to prevent recurrences. The first patient had diverticulosis with 1 ulcerated and inflamed diverticula at colonoscopy and underwent sigmoidectomy. Twenty-five months later, he developed a liver cyst infection recurrence. The second patient had partial hepatectomy and cholecystectomy for fistula between the liver bile duct and infected cyst, he was lost to follow-up 3 months after the surgery. The third patient had fecal transplantation for liver cyst infection recurrences associated with *Clostridium difficile* colitis; he had no recurrences 8 months after the transplantation. The fourth patient had an endoscopic bile duct extraction (bile duct lithiasis revealed on CT scan). She developed a liver cyst infection recurrence 1.5 months after the procedure. The fifth patient underwent endoscopic dilatation of common bile duct stenosis and did not develop recurrence after 6 months of follow-up. The sixth patient had liver transplantation for liver cyst infection recurrences.

## Discussion

The present study offers important insights into clinical presentation, microbiology, outcomes, and factors associated with treatment failure, relapse, and recurrence of liver cyst infection in patients with ADPKD. In contrast with previous studies describing and including cyst infections based on clinical and biological suspicion, suggestive signs at CT scan, MRI, or mostly ^18^FDG-PET/CT, were required in our study. This definition takes advantage of the now easier access to ^18^FDG-PET/CT, which is actually the most sensitive and specific imaging procedure to detect and localize cyst infection,^19^^,^^22^, ^23^, ^24^ thereby avoiding misdiagnosis in the presence of fever and abdominal pain (e.g. renal allograft pyelonephritis, colitis, or acute cholangitis).^12^ This definition allows us to distinguish between kidney and liver cyst infection, which are supposedly caused by different pathophysiology and causative microorganisms. Thus, our study might better reflect the factual outcomes of liver cyst infection as compared to previous studies.^6^^,^^15^^,^^25^

As previously described, *E coli* accounted for more than half of documented cyst infections^26^ with a susceptibility to third generation cephalosporins and fluoroquinolones in > 80% of cases and displayed a high rate of resistance (36%) to sulfamethoxazole/trimethoprim. When choosing a probabilistic antibiotic therapy, in addition to the microbiological history of the patient, the local antibiotic susceptibility patterns of microorganisms are important to consider, and our data should not be directly extrapolated to other countries. For example, in 1 center in Japan, fluoroquinolone susceptibility in cyst infections was extremely low (20%–30%).^8^ This is true at the regional level because we observed different levels of quinolone resistance between the 2 regions included in France (5% in Pays-de-La-Loire and 33% in Ile-de-France) (Supplementary Table S3). We suggest to use beta-lactam as the initial (probabilistic) antibiotic therapy in liver cyst infection given the frequent selection of resistant commensal,^27^ the increased risk of extended beta lactamase-producing pathogens,^28^ and the increasing rates of resistance with the use of quinolone and their class-specific side-effects.^29^ After clinical stabilization, empirical antibiotic treatment could be switched to fluoroquinolones provided that the cultured pathogen (when available) is confirmed to be susceptible to quinolones.

Treatment failure occurred in 14% of episodes of liver cyst infection, which is much lower than previously reported in the case-based review by Lantinga *et al.*^15^ (50%), partly because of the selection bias of this type of study as previously highlighted.^9^ We did not observe any deaths directly caused by liver cyst infection, in sharp contrast to a previous monocentric Japanese study,^16^ which reported 9.5% of deaths. However, the latter study suffered from a selection bias because patients with refractory liver cyst infection were referred to this single center from all over Japan. Early drainage of infected liver cysts to avoid treatment failure has been suggested in previous case series with small number of patients (*n* = 14 and *n* = 6).^6^^,^^17^ We could not agree with this recommendation because nearly all of our patients received antibiotics only and did not develop treatment failure. We introduced the notion of relapse (sometimes called early recurrence), which we analyzed in conjunction with treatment failure and should be distinguished from recurrences as suggested by a recent expert consensus.^20^ Treatment failure and relapse occurred in one-third of liver cyst infection episodes and were linked to a short duration of antibiotic therapy (< 2 weeks), in multivariable analysis. Cyst wall thickening has been associated with treatment failure in renal cyst infection^9^; however, we could not test this variable because wall thickness was rarely reported in our imaging reports.

Recurrence of liver cyst infection is associated with multiple hospitalizations, malnutrition, and chronic biological inflammatory syndrome; and sometimes requires major surgery such as partial hepatectomy or liver transplantation.^6^^,^^16^ Although several other studies^6^^,^^15^^,^^17^ have found a high rate of recurrence (more than one-third of cases in the present study), none have examined their risk factors, especially concerning the treatment modalities. After the first episode of liver cyst infection, there was a decreased risk of recurrence in patient with an antibiotic duration ≥ 28 days (OR = 0.12, 95% CI: 0.02–0.65, *P* = 0.021), consistent with our previous study on renal cyst infections and with current Kidney Disease: Improving Global Outcomes and the European Association for the Study of the Liver guidelines.^9^^,^^13^^,^^14^ Fluoroquinolone use was associated with a decreased risk of recurrence in univariate analysis; however, this association did not reach statistical significance in multivariable analysis. Because of their lipophilic properties, and supposedly better penetration into the cysts, fluoroquinolones are often recommended as the antibiotic of choice,^17^^,^^30^ replacing beta-lactams in the second line, mainly because of their lower diffusion capacity in uninfected cysts.^31^ However, we found no evidence for the added value of fluoroquinolone in both renal^9^ and liver cyst infections, probably because beta-lactams diffusion through the cyst wall is increased in case of inflammation, and their accumulation into the infected cysts could be obtained by a longer exposure time, as previously highlighted.^9^

In this study, follow-up ^18^FDG-PET/CT showed promising results in predicting relapse or recurrence of liver cyst infection. Although not statistically significant (*P* = 0.15), 6 of 8 (75%) and 3 of 9 patients (33%) with or without persistent liver uptake, respectively, have developed relapse or recurrent liver cyst infection within 12 months after the follow-up imaging. Persistent liver cyst hypermetabolism was defined by either no decrease or partial decrease of ^18^FDG uptake in the culprit cyst, suggesting that liver cyst infection has not been adequately treated, or resolution of ^18^FDG uptake in the former cyst but with the appearance of a new hypermetabolic cyst, suggesting a reinfection or contiguous infection. In our series, patients with persistent ^18^FDG uptake in the liver cyst at the time of follow-up imaging had a rather quiescent liver cyst infection, which could possibly remain asymptomatic for a certain period before developing a new sepsis (a recurrent episode). As highlighted below, recurrent episodes include both relapses and reinfections episodes, it is more likely that persistent cyst hypermetabolism at follow-up PET/CT is predictive of “late” relapses rather than reinfection. However, no decrease or partial decrease of ^18^FDG uptake in the cyst may be considered as a nonspecific healing process.^32^ Interestingly, in a previous series of 9 episodes of renal or liver cyst infections with follow-up ^18^FDG-PET/CT,^33^ we found no clear correlation between the persistence of hypermetabolic cysts and recurrence (1 recurrence for 6 episodes with persistent hypermetabolic cyst).^33^ These discrepancies could be partly explained by the inclusion of renal cyst infections, in this former study, which have a lower risk of recurrence (19%^9^ vs. 29% for liver cyst infection), thus, the event rate (recurrence) was supposed to be low, preventing us from drawing any definite conclusion about the usefulness of follow-up PET/CT at that time.

Attempts to reduce the risk of recurrence with antibiotic prophylaxis or invasive procedures to remove the presumed source of infection (e.g. polyps, diverticulosis, etc.) showed disappointing results. Even sigmoid colectomy to remove diverticulosis was tried in 1 patient, who nevertheless developed a new liver cyst infection shortly after surgery. The genesis of liver cyst infection is likely because of several interrelated factors such as digestive bacterial translocation into the portal vein and intrahepatic bile duct obstruction by liver cysts leading to microbiological pullulation. Therefore, antibiotic prophylaxis such as low dose cotrimoxazole is susceptible to inducing selection pressure, driving the evolution of bacterial resistance; and removal of colonic polyps, or even sigmoid colectomy is not sufficient because bile duct distortion persists. Nevertheless, it seems appropriate to use antibiotic prophylaxis to delay the onset of a new episode in selected patients with frequent liver cyst infection causing severe morbidity, such as malnutrition, and in those awaiting liver transplantation. Selective digestive tract decontamination is a topical antimicrobial agent that targets aerobic gram-negative pathogens in the gastrointestinal tract to prevent infection and is routinely used in intensive care units in the Netherlands. Bernts *et al.* reported 8 patients with polycystic liver disease and liver cyst infection who underwent selective digestive tract decontamination by oral polymyxin ± aminoglycoside.^34^ Although it showed promising results according to the authors, it induced severe side-effects in 2 patients, and bacterial resistance in 2 other patients. In addition, selective digestive tract decontamination to prevent infection has not been validated in countries with moderate to high prevalence of drug-resistant bacteria.^35^

We acknowledge several limitations in our retrospective study. Recurrence was defined by a new episode, 2 months after antibiotic discontinuation as recommended by an expert consensus^20^ which is a debatable definition because it did not discriminate between late relapse and reinfection (different pathogen). Positive CT scan, MRI, or ^18^FDG-PET/CT was a mandatory criterion for inclusion, this may have led to the exclusion of some cases of liver cyst infection. However, this is a strength of our study, because it avoids the inclusion of other abdominal infections, or cyst hemorrhages. Albeit statistically significant in univariable analysis, we did not find a protective effect of quinolone on treatment failure or relapses in multivariable analysis, larger studies are needed to confirm or refute these data. Data regarding liver volume and cyst wall thickening, both considered potential risk factors for recurrent episodes, and the management of immunosuppressive therapy in the kidney transplant recipient were not available. These factors may have influenced the risk of liver cyst infection relapse or recurrence. In patients with a history of kidney transplantation, recurrent liver cyst infections should prompt multidisciplinary discussion to reassess and potentially adjust immunosuppressive therapy. Finally, in France and Belgium, nephrologists usually manage renal but also hepatic cyst infections in ADPKD as part of the comprehensive patient care. Greater collaboration with hepatologists and microbiologists would help to better define consensual therapeutic guidelines.

Altogether, to the best of our knowledge, this study is the first to investigate risk factors associated with outcomes in liver cyst infection in patients with ADPKD, demonstrating the importance of prolonged antibiotic therapy to reduce the risk of relapse and recurrence. The latter being a major issue in patients with liver cyst infection. In our study, follow-up ^18^FDG-PET/CT appeared promising in predicting recurrence of liver cyst infection; its usefulness in guiding antibiotic therapy duration should be evaluated in a prospective trial.

## Disclosure

All the authors declared no competing interests.

## Supplementary Material

Supplementary File (PDF) **Figure S1.** Imaging of initial and follow-up 18FDG-PET/CT. Table S1. Performance of CT scan or ^18^FDG-PET/CT to detect liver cyst infection. **Table S2.** Detailed microbiological data of the 70 documented liver cyst infections. **Table S3.** Susceptibility of *Escherichia coli* to main empirical antibiotics use in liver cyst infection in 4 centers in Belgium and France. **Table S4.** Detailed prophylaxis used in patients with recurrent liver cyst infections.

## Footnotes

Supplementary File (PDF)
**Figure S1.** Imaging of initial and follow-up 18FDG-PET/CT. **Table S1.** Performance of CT scan or ^18^FDG-PET/CT to detect liver cyst infection. **Table S2.** Detailed microbiological data of the 70 documented liver cyst infections. **Table S3.** Susceptibility of *Escherichia coli* to main empirical antibiotics use in liver cyst infection in 4 centers in Belgium and France. **Table S4.** Detailed prophylaxis used in patients with recurrent liver cyst infections.
