# Supplementary material for: Liver Cyst Infection Outcomes in Patients With ADPKD
Source: Kidney Int Rep. 2025 Nov 4;11(1):94–105. doi: 10.1016/j.ekir.2025.10.027 (PMC12799584; doi:10.1016/j.ekir.2025.10.027)
Supplement: Supplementary File (PDF) — Figure S1. Imaging of initial and follow-up 18FDG-PET/CT. Table S1. Performance of CT scan or 18FDG-PET/CT to detect liver cyst infection. Table S2. Detailed microbiological data of the 70 documented liver cyst infections. Table S3. Susceptibility of Escherichia coli to main empirical antibiotics use in liver cyst infection in 4 centers in Belgium and France. Table S4. Detailed prophylaxis used in patients with recurrent liver cyst infections. [file mmc1.pdf]

Supplemental material

Supplemental Table S1. Performance of CT-scan or ^{18}FDG-PET/CT to detect liver cyst infection

Imaging exam performance	Definite liver cyst infection n = 18
**Positive CT-Scan / CT-scan performed**	5/13 (39%)[a]
**Positive ^{18}FDG-PET/CT / ^{18}FDG-PET/CT performed**	13/14 (93%)[b]

[a] 7/13 contrast-enhanced CT-scan

[b] PET-CT was negative in 1 episode while MRI was suggestive of liver cyst infection

Supplemental Table S2. Detailed microbiological data of the 70 documented liver cyst infections

**Microbiological data of documented liver cyst infections**	**Documented liver cyst infection episode n=70**
**Polymicrobial**[a]	5/70 (7%)
**Enterobacteriaceae**	57/70 (81%)
Escherichia coli	36/70 (51%)
Klebsiella pneumoniae	10/70 (14%)
Klebsiella oxytoca	3/70 (4%)
Klebsiella aerogenes	3/70 (4%)
Klebsiella variicola	1/70 (1%)
Citrobacter freundii	2/70 (3%)
Serratia marcescens	1/70 (1%)
Raoultella species	3/70 (4%)
**Non-fermenting gram-negative bacilli**	9/70 (13%)
Pseudomonas aeruginosa	7 (10%)
Pseudomonas fluorescens	1 (1%) [b]
Acinetobacter spp	2 (3%)
**Gram positive**	8/70 (11%)
Enterococcus, n (%)	3/70 (4%)
E. faecium	2/70 (3%)
E. gallinarum	1/70 (1%)
Staphylococcus, n (%)	3/70 (4%)
S. epidermidis	2/70 (3%)
S. hominis	1/70 (1%)
Lactobacillus, n (%)	1/70 (1%)
Streptococcus pneumoniae, n (%)	1/70 (1%)
**Fungi**[c]	1/70 (1%)

Continuous variables were described as mean and SD, and categorical variables were described as counts and percentages.

[a] *Pseudomonas aeruginosa + Enterobacter complex cloacae, E. coli + Pseudomonas aeruginosa + Pseudomonas fluorescens, E.coli + E. faecium, E.coli + Citrobacter freundii + Klebsiella pneumoniae* and *E.coli + Acinetobacter* species.

[b] *Pseudomonas aeruginosa + Pseudomonas fluorescens*

[c] *Candida glabrata*.

Supplemental Table S3. Susceptibility of *Escherichia coli* to main empirical antibiotics used in liver cyst infection in 4 centres in Belgium and France (Wallonia n=1, Ile de France n=2 and Pays de la Loire n=1)

	Resistant (n)	Susceptible (n)	Total (n)	Sensitivity (%)
**Quinolones**	5	27	32	84%
**Third generation cephalosporin**	4	28	32	88%
**Amoxicillin-clavulanic acid**	6	26	32	81%
**Piperacillin-Tazobactam**	3	29	32	91%
**Sulfamethoxazole- Trimethoprim**	11	21	32	66%

Of the 18 available antibiotic susceptibility testings for *E. coli* in Pays de la Loire 1 was quinolone resistant (5%), none was third generation cephalosporin resistant, 1 (5%) was amoxicillin-clavulanic acid resistant, 0 was piperacillin-tazobactam resistant and 3 (17%) was sulfamethoxazole-trimethoprim resistant). Of the 9 available antibiotic susceptibility testings for *E. coli* in Ile de France, 3 (33%) was quinolone resistant, 4 (44%) was third generation cephalosporin resistant, 5 (56%) was amoxicillin-clavulanic acid resistant, 2 (22%) was piperacillin-tazobactam resistant and 8 (89%) was sulfamethoxazole-trimethoprim resistant. In the 5 available antibiotic susceptibility testings for *E. coli* in Wallonne, 1 (20%) was quinolone resistant, 1 (20%) was third generation cephalosporin resistant, 1 (20%) was amoxicillin-clavulanic acid resistant, 1 (20%) was piperacillin-tazobactam resistant and 1 (20%) was sulfamethoxazole-trimethoprim resistant.

Supplemental Table S4. Detailed prophylaxis used in patients with recurrent liver cyst infections

Patients	Age/ Gender	Non-invasive prophylaxis		
		**Number of liver cyst infections before the start of prophylaxis**	**Type of prophylaxis**	**Liver cyst infection recurrence/Delay (months)**
1	75/F	7	Fosfomycin/Cotrimoxazole (cycling)	Yes/ 2 months after the start of antibiotic cycling.
2	53/F	5	1st generation Cephalosporin (Cephalexin)	No after 4.5 months of prophylaxis (liver transplant waiting list).
3	76/M	5	Cotrimoxazole	No after 6 months of prophylaxis.
4	65/F	1	Fluoroquinolone	No after 1.5 months of prophylaxis (liver transplant waiting list).
5	81/F	1	Oral third generation cephalosporin (Cefixime) and Fosfomycin (cycling)	No after 12 months of prophylaxis.
6	50/F	1	Oral third generation cephalosporin (Cefixime) and Fosfomycin + E.coli vaccines and E.coli bacteriophage therapy	No after 73 months of prophylaxis.
7	71/F	1	Amoxicillin + oral 3rd generation cephalosporin (Cefixime) + Moxifloxacin (cycling)	Yes/ 4 months.
7	74/F	6	Intra-venous immunoglobulin	Yes/11 months.
7	75/F	7	Fosfomycin + Pivmecillinam (cycling)	Yes/9 months.
8	79/M	3	Cotrimoxazole	No after 8 months of prophylaxis.
**Patients**	**Age/ Gender**	**Invasive procedure to prevent liver cyst infection**		
		**Number of liver cyst infections before the start of prophylaxis**	**Type of invasive procedure**	**Liver cyst infection recurrence/Delay (months)***
1	52/M	1	Sigmoid colectomy (diverticulosis)	Yes/25.5 months.
2	60/F	1	Partial hepatectomy and cholecystectomy (fistula between liver bile duct and infected cyst)	No after 3 months of follow-up.
3	59/F	1	Liver transplantation	No.
4	54/F	5	Fecal transplantation	No (each episode of liver cyst infection was associated with clostridium difficile colitis) after 8 months of the fecal transplantation.

5	73/F	3	Endoscopic bile duct stone extraction	Yes/1.5 months after the endoscopic procedure.
6	79/F	1	Endoscopic biliary stenosis dilatation	No after 6 years of follow-up.

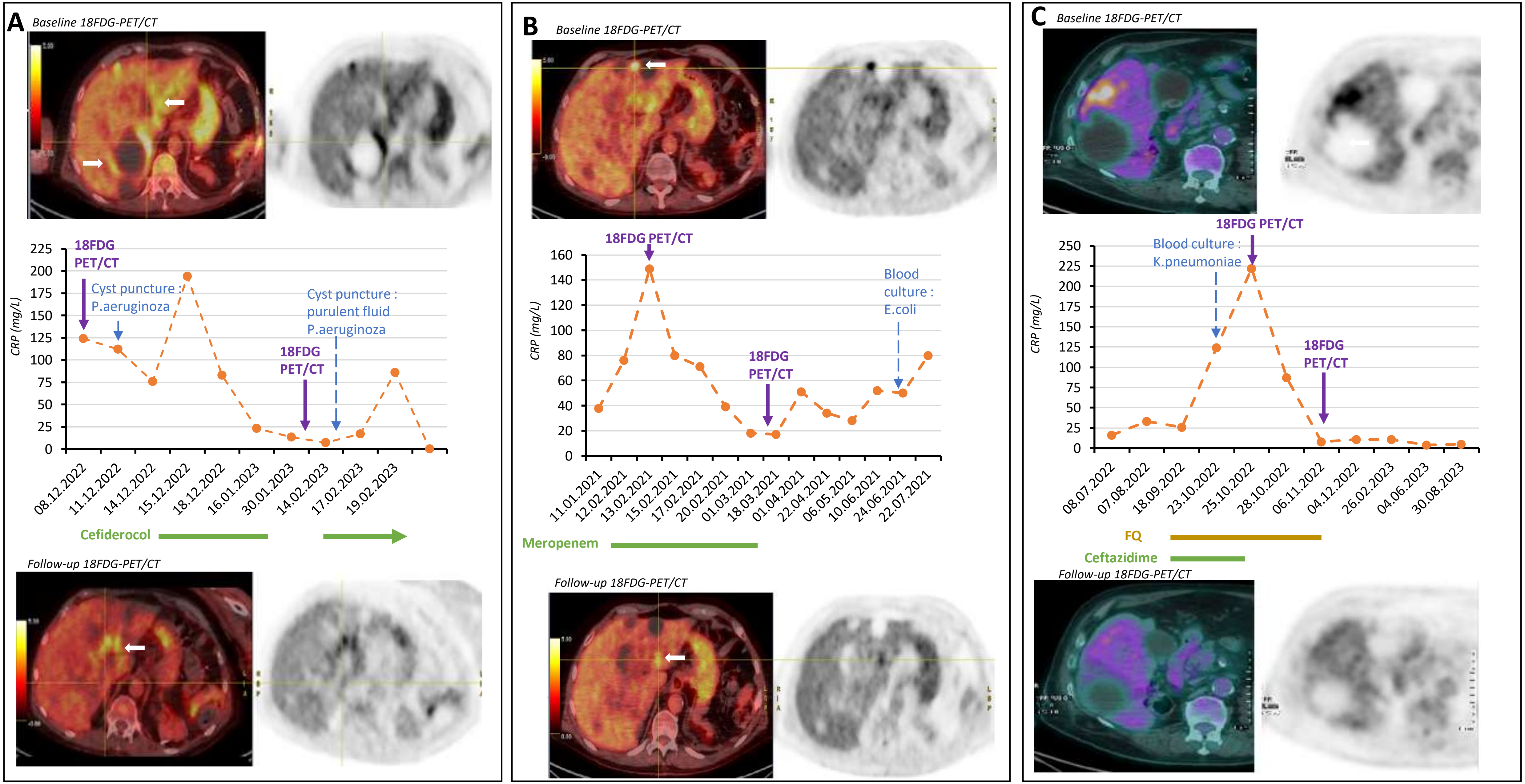

A
Baseline 18FDG-PET/CT
CRP (mg/L)
18FDG PET/CT
Cyst puncture : P.aeruginoza
Cyst puncture : purulent fluid P.aeruginoza
18FDG PET/CT
08.12.2022
11.12.2022
14.12.2022
15.12.2022
18.12.2022
16.01.2023
30.01.2023
14.02.2023
17.02.2023
19.02.2023
Cefiderocol
Follow-up 18FDG-PET/CT
B
Baseline 18FDG-PET/CT
CRP (mg/L)
18FDG PET/CT
18FDG PET/CT
Blood culture : E.coli
11.01.2021
12.02.2021
13.02.2021
15.02.2021
17.02.2021
20.02.2021
01.03.2021
18.03.2021
01.04.2021
22.04.2021
06.05.2021
10.06.2021
24.06.2021
22.07.2021
Meropenem
Follow-up 18FDG-PET/CT
C
Baseline 18FDG-PET/CT
CRP (mg/L)
18FDG PET/CT
Blood culture : K.pneumoniae
18FDG PET/CT
08.07.2022
07.08.2022
18.09.2022
23.10.2022
25.10.2022
28.10.2022
06.11.2022
04.12.2022
26.02.2023
04.06.2023
30.08.2023
FQ
Ceftazidime
Follow-up 18FDG-PET/CT
